# Operation for Imperforate Anus

**Published:** 1841-06-19

**Authors:** J. Pancoast

**Affiliations:** Professor of Anatomy in Jefferson Medical College


*Operation for Imperforate Anus.* By J. Pancoast, M. D., Professor of Anatomy in Jefferson Medical College.

*June 2d*, 1841.—Dr. Pancoast met in consultation Dr. Johnson, a distinguished practitioner of Norristown, in this state, on the case of the infant son of a gentleman of that place, born with imperforate anus. The infant was five days old, and otherwise healthy and well formed. It took nourishment as in ordinary cases, and had been troubled only during the last twenty-four hours with vomiting, when it ejected merely the contents of the stomach. It had latterly become fretful and restless, especially during the night. On examining the anal region, the usual fissure was found existing between the buttocks. At the point of the os coccygis and behind the natural position of the anus, there was a small cup like depression. An inch and a half in front of this depression there was a small elevated crest, shaped like the caput gallinaginis. The intervening space was flat and firm, though a sense of deep fluctuation could be felt when pressed on with the finger. In the crest, which was above the natural position of the anus, there was a minute, imperceptible orifice, from which two or three drachms of meconium had in all been at various times forced out by the straining of the child. An anal opening was made by plunging in a straight bistoury an inch long in the blade, and dividing, along the median line, the parts for an inch in the direction of the os coccygis. About five gills of feted meconium were discharged, with entire relief to the child. The rectum had terminated in a *cul de sac*, above the internal sphincter.

